# Inhibition of cell migration by ouabain in the A549 human lung cancer cell line

**DOI:** 10.3892/ol.2013.1406

**Published:** 2013-06-17

**Authors:** NING LIU, YALI LI, SUWEN SU, NA WANG, HUICI WANG, JUNXIA LI

**Affiliations:** 1 The Key Laboratory of Pharmacology and Toxicology for New Drugs, Department of Pharmacology, Hebei Medical University;; 2 The Key Laboratory of Neural and Vascular Biology, Ministry of Education, Hebei Medical University, Shijiazhuang, Hebei 050017;; 3 Hebei Provincial People’s Hospital, Shijiazhuang, Hebei 050051, P.R. China

**Keywords:** ouabain, cancer, epithelial-mesenchymal transition, migration, Na^+^/K^+^-ATPase inhibitor

## Abstract

The Na^+^/K^+^-ATPase α subunit is highly expressed in malignant cells. Ouabain, a cardioactive glycoside, binds to the Na^+^/K^+^-ATPase α subunit and inhibits the activity of Na^+^/K^+^-ATPase. In the present study, the effect of ouabain on the migration of A549 cells was analyzed using the wound healing and transwell chamber migration assays. The impact of ouabain on the expression of E-cadherin, N-cadherin, vimentin, matrix metalloprotease (MMP)-2 and MMP-9 was also evaluated. Ouabain treatment not only inhibited the epidermal growth factor (EGF)-enhanced migration of A549 cells, but also inhibited the basal migration of A549 cells in the absence of EGF. Ouabain decreased the overexpression of N-cadherin and vimentin induced by EGF, and decreased the expression of MMP-2 and -9 in the presence or absence of EGF. Na^+^/K^+^-ATPase is a potent therapeutic target in lung cancer and these observations indicated that the Na^+^/K^+^-ATPase inhibitor, ouabain, retards the invasion of lung cancer cells.

## Introduction

Lung cancer is one of the major causes of cancer-related mortality worldwide. Despite extensive studies concerned with diagnostic and treatment strategies, the 5-year survival rate for lung cancer patients is only 10% (1,2). Na^+^/K^+^-ATPase, a sodium pump, has been hypothesized to represent a new target for the development of anticancer drugs. Na^+^/K^+^-ATPase is responsible for the regulation of ion homeostasis in mammalian cells by exporting three Na^+^ in exchange for two K^+^. Na^+^/K^+^-ATPase is involved in cancer progression and is significant in cell adhesion and signaling (3,4). Cardiac glycosides, which inhibit Na^+^/K^+^-ATPase, have been used for treating arrhythmia and congestive heart failure for >200 years (5). Previous epidemiological studies have implied that cardiac glycosides are capable of inhibiting the growth of cancer cells and decreasing the incidence of certain types of tumors, including leukemia, lymphoma, kidney tumors and urinary tumors. Tumor metastasis is one of the main features of malignant cancer and is the main cause of mortality in cancer patients (6,7).

Tumors derived from the epithelium comprise 90% of all human malignant tumors. A developmental regulatory process, referred to as epithelial-mesenchymal transition (EMT), is prominently implicated in the ability of transformed epithelial cells to disseminate and become resistant to apoptosis (8–10). During EMT, epithelial cells often lose their primary characteristics and adopt the characteristics of mesenchymal cells by losing their polarity and increasing invasion capabilities. EMT may occur in a number of types of epithelial tumors, including lung, breast, pancreatic and gastric cancer (11–13). Non-small cell lung cancer (NSCLC) is the predominant type of lung cancer and accounts for ∼80% of all lung cancer cases (14). The A549 cancer cell line, which has features of type II alveolus epithelial cells, is a human NSCLC cell line (15). Anoxia, epidermal growth factor (EGF) and transforming growth factor (TGF)-β all induce EMT (16). During EMT, the expression of N-cadherin and vimentin increases, and the expression of E-cadherin decreases (17). Matrix metalloprotease (MMP)-2 and MMP-9 are involved in cancer cell adhesion, invasion and migration, and have been hypothesized to represent prognostic biomarkers for lung cancer progression. In the present study, the effect of the Na^+^/K^+^-ATPase inhibitor, ouabain, on the migration of A549 cells was detected.

## Materials and methods

### Drugs and reagents

The following drugs and reagents were used in this study: Ouabain (Sigma-Aldrich, St. Louis, MO, USA); RPMI-1640 medium (Gibco-BRL, Carlsbad, CA, USA); transwell chambers (Corning Inc., Corning, NY, USA); mitomycin C (Sigma-Aldrich); trypase (Gibco-BRL); and rabbit antibodies against MMP-2, MMP-9, N-cadherin, E-cadherin and vimentin (Proteintech, Chicago, IL, USA).

### Cell lines

The A549 human NSCLC cell line (Institute of Cell Biology, Chinese Academy of Science, Shanghai, China) was grown in RPMI-1640 supplemented with 10% fetal bovine serum, 100 *μ*g/ml penicillin and 100 *μ*g/ml streptomycin at 37°C in a 5% CO_2_ humidified atmosphere.

### Wound healing assay

A549 cells that had been treated with the control medium or medium containing EGF for 48 h were seeded in 24-well cell culture plates and cultured to a confluent monolayer. A pipette tip (10 *μ*l) was used to scratch a wound on the midline of the culture well, and the cells were pretreated with 10 *μ*g/ml mitomycin C for 1 h at 37°C; the cells were then washed twice with PBS. Following 15 h of culture in RPMI-1640 supplemented with 2% serum (control) or other stimulants, the migration of the cells was evaluated by measuring the difference in the area of the wounds with a Leica DM2500 image analysis system (Leica, Mannheim, Germany) at 0 and 15 h.

### Transwell chamber migration assay

A549 cells (2×10^4^) that had been treated with the control medium or with medium containing EGF for 48 h were added to transwell chambers. Serum was added to the bottom wells of the chambers to induce cell migration. After 15 h, the cells that had migrated through the membrane were stained with 0.5% methylrosaniline chloride solution and counted. Cells were counted in five random fields and expressed as 1% of the average number of cells/field under a light microscope.

### Western blot analysis

Whole cell extracts of the cancer cells were prepared using a whole cell extraction kit (Beyotime, Nantong, China). In brief, cells were washed with cold PBS and scraped free in the presence of lysis buffer (20 mmol/l MOPS, 2 mmol/l EGTA, 5 mmol/l EDTA, 30 mmol/l NaF, 40 mmol/l β-glycerophosphate, 20 mmol/l sodium pyruvate, 0.5% Triton X-100 and 1 mmol/l sodium orthovanadate with protease inhibitor cocktail). The lysates were then put on ice for 30 min prior to centrifugation at 12,000 × g for 30 min at 4°C. Protein samples were resolved by 10% SDS-PAGE and electrotransferred to polyvinylidene fluoride (PVDF) membranes in transfer buffer (20% methanol, 15.6 mM Tris base and 120 mM glycine), according to standard methods. Following a 2-h incubation in 5% non-fat dry milk blocking buffer prepared in TBS with 0.1% Tween-20, the PVDF membranes were incubated for 15 h at 4°C with a primary antibody diluted to 1:200–1:500 in blocking buffer. Following three TBST buffer washes for 10 min/time, the membranes were then incubated with anti-rabbit or anti-mouse secondary antibodies conjugated to IRDye 700DX or 800CW (1:5,000) for 2 h at 37°C. Protein bands were detected and quantified on a two-color Odyssey Infrared Imaging System (LI-COR Biosciences, Lincoln, NE, USA).

### Data analysis and statistics

Data are presented as the mean ± SEM. All experiments were repeated at least three times. Differences were analyzed with the one-way ANOVA with SPSS 13.0 software (SPSS, Inc., Chicago, IL, USA). P<0.05 was considered to indicate a statistically significant difference.

## Results

### EGF induces morphological changes in A549 cells

**Figure 1.**
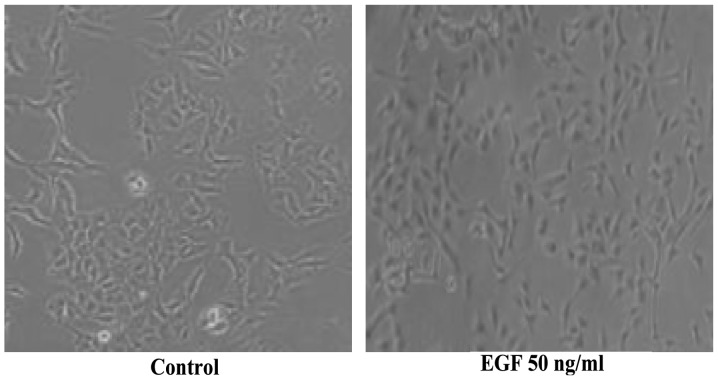
Effect of 50 ng/ml EGF on the morphology of the A549 cell line. EGF, epidermal growth factor.

Morphological changes were observed in A549 cells treated with 50 ng/ml EGF; these cells became isolated and certain pseudopods were stretched out. In addition, the intercellular tight junctions became loose and the shape of the cells changed from being cobblestone-like to fibroblast-like in appearance. These characteristic changes indicated that EGF had induced EMT in the A549 cells (Fig. 1).

### Effect of ouabain on the migration of the A549 cell line

**Figure 2.**
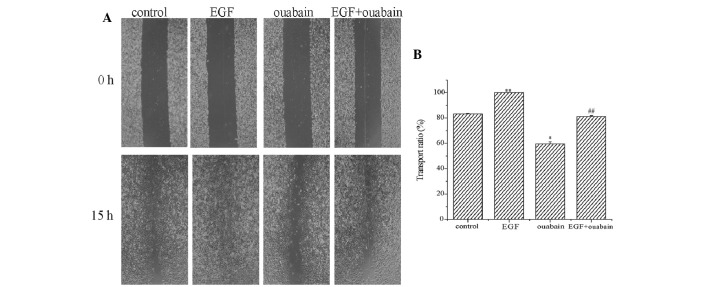
Ouabain inhibits EGF-enhanced and basal migration of A549 cells. (A) Wound healing assay to determine the effect of ouabain on A549 cell migration. (B) The summarized migration ratio (%) within 15 h measured by wound healing assay. Each column represents the mean ± SEM. ^*^P<0.05 and ^**^P<0.01, vs. control; ^##^P<0.01, vs. EGF. EGF, epidermal growth factor.

A wound healing assay was performed to observe the effect of ouabain on the migration of A549 cells. To exclude the growth inhibitory effect of ouabain on migration, the concentration of ouabain used was 25 nM, which did not affect the growth of cancer cells for 15 h. The effect of 25 nM ouabain on the expression of PCNA in the A549 cells for 15 h was detected, and it was identified that ouabain did not affect the expression of PCNA. The A549 cells incubated in RPMI-1640 medium with or without EGF (50 ng/ml) for 48 h were used in the experiment. The results of the wound healing assays are presented in Fig. 2. The area that the cells had migrated within 15 h (toward the initially scratched midline, from the border line) was measured. The cells incubated with 50 ng/ml EGF migrated across an area that was larger than that of the cells incubated with the control medium, indicating that EGF stimulated the migration of the A549 cells. The migration ratio was 83% in the control group and almost 100% in the EGF group. Following 15 h of treatment, ouabain not only inhibited EGF-enhanced migration, but also inhibited basal migration of the A549 cells in the absence of EGF. Ouabain decreased the migration ratio from 83 to 59% in the absence of EGF and from ∼100 to 82% in the presence of EGF.

**Figure 3.**
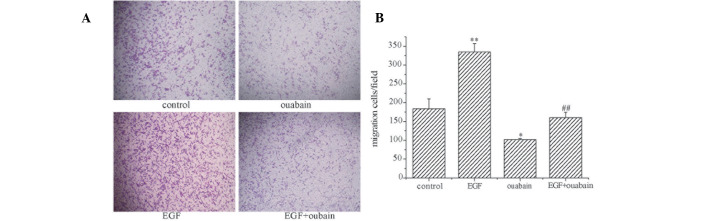
Ouabain inhibits EGF-enhanced and basal migration of A549 cells. (A) Effect of ouabain on A549 cell migration using the transwell assay. Staining, 0.5% methylrosaniline chloride solution; magnification, ×20. (B) Quantification of migrating cells. Each column represents the mean ± SEM. ^*^P<0.05 and ^**^P<0.01, vs. control; ^##^P<0.01, vs. EGF. EGF, epidermal growth factor.

To further investigate the role of ouabain in the migration of the A549 cells, a transwell chamber migration assay was performed to detect the effect of ouabain on the number of migrating cells. The results of the transwell assay were consistent with those of the wound healing assay (Fig. 3).

### Effect of ouabain on the expression of E-cadherin, N-cadherin and vimentin

E-cadherin, N-cadherin and vimentin are the main biomarkers of EMT. In this study, the expression of E-cadherin, N-cadherin and vimentin was detected following EGF treatment. EGF significantly increased the expression of vimentin in A549 cells, marginally (but significantly) increased the expression of N-cadherin and exerted no effect on the expression of E-cadherin. The enhancement of vimentin and N-cadherin indicated that EMT occurred in the A549 cells following EGF treatment. These results revealed that EGF induced EMT in the A549 cells, and that EMT may be significant in EGF-induced migration of A549 cells.

**Figure 4.**
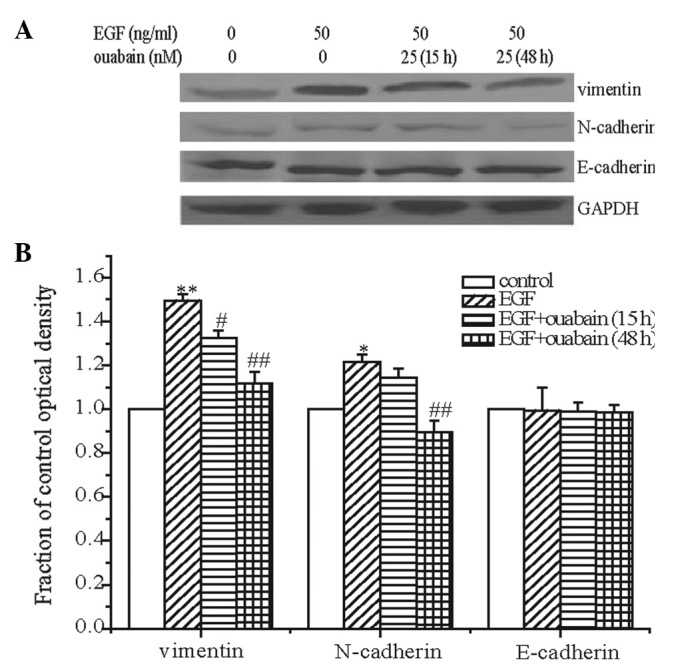
Effect of ouabain on the expression of E-cadherin, N-cadherin and vimentin in the A549 cell line. (A) Representative western blotting results. (B) Quantitative data from three independent experiments. Each column represents the mean ± SEM. ^*^P<0.05 and ^**^P<0.01, vs. control; ^#^P<0.05 and ^##^P<0.01, vs. EGF. EGF, epidermal growth factor.

Treatment with ouabain for 15 h decreased the expression of vimentin and had no clear effect on the expression of N-cadherin. The expression of vimentin and N-cadherin was further detected following ouabain treatment for 48 h. The results revealed that ouabain decreased the expression of vimentin and N-cadherin. Ouabain had no effect on the expression of E-cadherin (Fig. 4).

### Effect of ouabain on the expression of MMP-2 and -9

**Figure 5.**
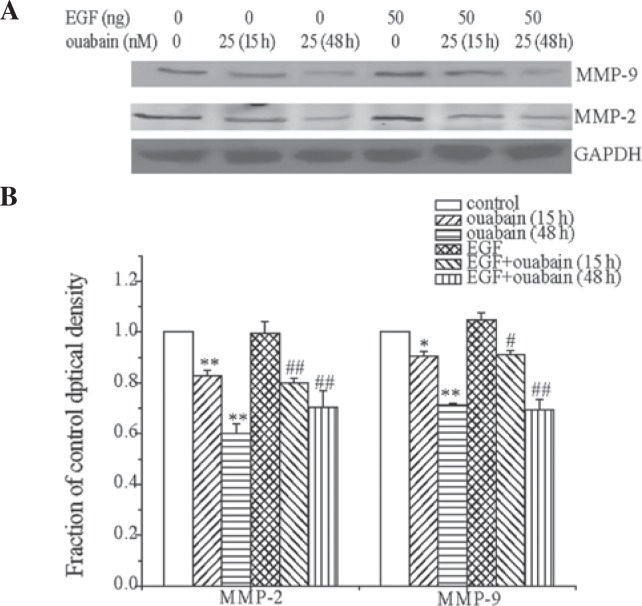
Effects of treatment with 25 nM ouabain for 15 and 48 h on the expression of MMP-2 and -9 in the A549 cell line. (A) Representative western blotting results. (B) Quantitative data from three independent experiments. Each column represents the mean ± SEM. ^*^P<0.05 and ^**^P<0.01, vs. control; ^#^P<0.05 and ^##^P<0.01, vs. EGF. MMP, matrix metalloproteinase; EGF, epidermal growth factor.

MMP-2 and -9 degrade the intercellular mesenchyme and promote tumor migration. Expression of MMP-2 and -9 was detected following treatment with ouabain for 15 h. The results demonstrated that ouabain downregulated the expression of MMP-2 and -9 in the presence or absence of EGF. In addition, the expression of MMP-2 and -9 was further detected following treatment with ouabain for 48 h. Similarly, the results revealed that ouabain decreased the expression of MMP-2 and -9 in the presence or absence of EGF (Fig. 5).

## Discussion

EMT describes a series of rapid changes in the cellular phenotype. During EMT, epithelial cells downmodulate their cell-cell adhesion structures, alter their polarity, reorganize their cytoskeleton, become resistant to apoptosis and acquire the capacity for differentiation. EMT is tightly regulated under normal physiological circumstances; however, in tumor tissues, normal regulation is lost, which results in EMT and provides the cancer cells with the capacity for migration (18).

Numerous factors, including anoxia, TGF-β, VEGF and EGF, induce EMT. In the present study, 50 ng/ml EGF was used to induce EMT in the A549 cells. Following EGF treatment, the morphology of the A549 cells became similar to that of mesenchyme cells, the intercellular joints became loose and the cells became isolated with stretched out pseudopods. Furthermore, the results of the wound healing and transwell chamber migration assays revealed that the migration velocity was increased following EGF treatment. All results demonstrated that EMT occurred following EGF treatment, and the expression of N-cadherin, E-cadherin and vimentin was detected. One of the indications of EMT is cadherin transformation, which is defined by a decrease in E-cadherin and an increase in N-cadherin expression. Occasionally, E-cadherin expression does not change and only that of N-cadherin increases (19,20). E-cadherin is a calcium-dependent transmembrane glycoprotein that is important for intercellular tight junctions in epithelial cells. Vimentin is a skelemin protein that is highly expressed in mesenchymal cells and rarely expressed in epithelial cells. Vimentin is important for maintaining the structure and function of interstitial cells (21). In the present study, following EGF treatment, the expression of E-cadherin did not change, the expression of N-cadherin marginally (but significantly) increased and the expression of vimentin significantly increased. These results indicated that EGF induced EMT in A549 cells. The effect of ouabain on the expression of E-cadherin, N-cadherin and vimentin was also detected. The results revealed that ouabain lowered the expression of N-cadherin, and vimentin and had no effect on the expression of E-cadherin.

The wound healing and transwell chamber migration assay results demonstrated that ouabain decreased the migration velocity of the A549 cells. Uddin *et al* identified that the Na^+^/K^+^-ATPase inhibitor, marinobufagenin, inhibited the proliferation, invasion and migration of cytotrophoblasts via the ERK1/2 signaling pathway (22). Huang *et al* reported that the Na^+^/K^+^-ATPase inhibitor, ursolic acid, inhibited the invasion and migration of lung cancer cells by decreasing the activity of VEGF, MMP and ICAM-1 (23). Therefore, the present study concluded that the effect of ouabain on A549 cells may have been due to the inhibition of Na^+^/K^+^-ATPase activity. MMPs, which are secreted from tumor and/or stroma cells, are involved in tumor invasion and metastasis by degrading the extracellular matrix surrounding the tumor, particularly the basement membrane (24). The majority of previous studies have demonstrated that high protein expression levels of MMP-2 and -9 correlate with a poorer prognosis (25). In the present study, it was indicated that ouabain downregulated the expression of MMP-2 and -9. Therefore, the effect of ouabain on A549 migration may, in part, be due to the effect of ouabain on MMP-2 and -9. In conclusion, this study demonstrates that the Na^+^/K^+^-ATPase inhibitor ouabain retards the migration of A549 cells. The results suggest that Na^+^/K^+^-ATPase may be a potential therapeutic target in lung cancer.
